# Activation of the AMP-Activated Protein Kinase by Eicosapentaenoic Acid (EPA, 20:5 n-3) Improves Endothelial Function *In Vivo*

**DOI:** 10.1371/journal.pone.0035508

**Published:** 2012-04-19

**Authors:** Yong Wu, Cheng Zhang, Yunzhou Dong, Shuangxi Wang, Ping Song, Benoit Viollet, Ming-Hui Zou

**Affiliations:** 1 Division of Endocrinology and Diabetes, Department of Medicine, University of Oklahoma Health Science Center, Oklahoma City, Oklahoma, United States of America; 2 Département de Génétique, Développement et Pathologie Moléculaire, Institut Cochin, Université René Descartes Paris 5, Institut National de la Santé et de la Recherche Medicale U567, Centre National de la Recherchè Scientifique UMR8104, Paris, France; The Chinese University of Hong Kong, Hong Kong

## Abstract

The aim of the present study was to test the hypothesis that the cardiovascular-protective effects of eicosapentaenoic acid (EPA) may be due, in part, to its ability to stimulate the AMP-activated protein kinase (AMPK)-induced endothelial nitric oxide synthase (eNOS) activation. The role of AMPK in EPA-induced eNOS phosphorylation was investigated in bovine aortic endothelial cells (BAEC), in mice deficient of either AMPKα1 or AMPKα2, in eNOS knockout (KO) mice, or in Apo-E/AMPKα1 dual KO mice. EPA-treatment of BAEC increased both AMPK-Thr172 phosphorylation and AMPK activity, which was accompanied by increased eNOS phosphorylation, NO release, and upregulation of mitochondrial uncoupling protein-2 (UCP-2). Pharmacologic or genetic inhibition of AMPK abolished EPA-enhanced NO release and eNOS phosphorylation in HUVEC. This effect of EPA was absent in the aortas isolated from either eNOS KO mice or AMPKα1 KO mice fed a high-fat, high-cholesterol (HFHC) diet. EPA via upregulation of UCP-2 activates AMPKα1 resulting in increased eNOS phosphorylation and consequent improvement of endothelial function *in vivo*.

## Introduction

Eicosapentaenoic acid (20:5Delta (5,8,11,14,17); ω-3: EPA) is an ω-3 polyunsaturated fatty acid (PUFA), which is abundant in fish oils. Epidemiological and clinical trials have shown that ω-3 fatty acids, in particular EPA, reduce cardiovascular deaths [1] and retard the progression of atherosclerosis in coronary patients [2]. The precise mechanism by which fish oils inhibit atherosclerosis is still unclear, but it may relate to the modulation of lipid metabolism [3], improvement of vascular endothelial function [4], enhancement of vascular reactivity and compliance [5], reduction of cytokine production [6], and inhibition of inflammatory processes [7].

Nitric oxide (NO) is essential for endothelial function. Decreased NO bioactivity is involved in the pathogenesis of many cardiovascular disorders such as hypertension, atherosclerosis, venous bypass graft disease, diabetic vascular disease [8]. There is evidence that the beneficial effects of EPA may be due to its ability to augment levels of NO. In diabetic rats, long term oral administration of EPA may stimulate NO production, and increased NO levels likely inhibit enhanced cardiac sympathetic activity in these animals [9]. Similarly, the n-3 fatty acids promote the synthesis of beneficial NO in the endothelium [10]. In rabbits, EPA reduces myocardial infarct size, primarily through calcium channel–mediated mechanisms and partially through NO-mediated mechanisms [11]. Accumulating evidence shows that ω-3 PUFA can regulate NOS activity and increase NO synthesis in endothelial cells and vascular smooth muscle cells [12], [13]. However, the mechanisms underlying ω-3 PUFA-enhanced NO release remain poorly understood.

The AMP-activated protein kinase (AMPK) is a heterotrimeric protein composed of α, β, and γ subunits. The α subunit imparts catalytic activity, while the β subunit contains a glycogen-binding domain (GBD) that also regulates the activity and the γ subunit forms the broad base of the protein and is required for AMP binding [14], [15]. AMPK is well-conserved among eukaryotic cells and is expressed by endothelial cells of different origins [16], [17], [18]. Activation of AMPK requires phosphorylation of Thr172 in the activation loop of the α subunit [19] and is mediated by at least two kinases, Peutz-Jeghers syndrome kinase LKB1 [20] and Ca^2+^/calmodulin-dependent protein kinase kinase (CaMKK) [21]. AMPK has been shown to mediate angiogenic and anti-inflammatory effects, which are thought to be due to NO formation [17], [22], [23], [24]. AMPK is reported to phosphorylate endothelial nitric oxide synthase (eNOS) at Ser1177 or 1179 [17], [22] and enhance the interaction between eNOS and heat shock protein 90 [25]. Based on these reports, we hypothesized that the protective effects of EPA on the cardiovascular system may be due, in part, to the ability of EPA to stimulate AMPK-induced eNOS activation and consequently, NO production. Here, we provide evidence of a novel pathway in which EPA activates AMPK in endothelial cells through an upregulation of UCP-2.

## Results

### EPA induces AMPK phosphorylation and activation

**Figure 1:**
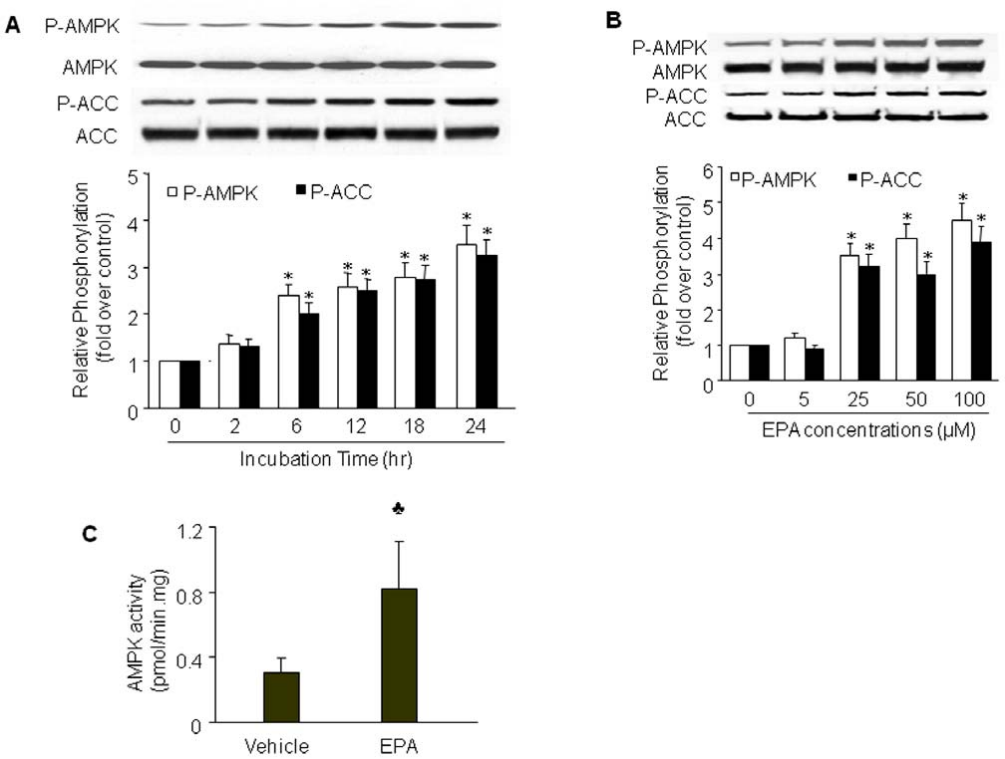
EPA activates AMPK in BAEC. BAEC were treated with (**A**) 25 µmol/L EPA for the indicated times or (**B**) varying concentrations of EPA for 24 h. Lysates (80 µg) were analyzed by western blot for the indicated proteins. The blot is a representative of four blots obtained from four separate experiments. Corresponding densitometric analyses of phosphorylated AMPK and ACC are shown. *, *P*<0.05 *vs.* control groups. **C**) Confluent BAECs were treated with vehicle or EPA (25 µmol/L) for 24 hours. AMPK activity was assayed using the SAMS peptide as a substrate. Data presented are means ± SD from 3 independent experiments. *P*<0.05 *vs.* vehicle.

To investigate whether EPA activates AMPK in endothelial cells, confluent BAEC or HUVEC were treated with varying concentrations of EPA for 2 to 24 h. AMPK activation was indirectly assessed by western blot analysis of AMPK phosphorylation at Thr172, which is essential for AMPK activity [26]. Ser79 phosphorylation of ACC, a substrate of AMPK [27], was also used as an indicator of AMPK activation. As shown in Figure 1A, the phosphorylation of both AMPK and ACC gradually increased beginning from 6 h after incubation with 25 µM of EPA and reached peak levels at 24 h in BAEC. Increased AMPK phosphorylation was associated with elevated AMPK activity, as measured by the SAMS peptide assay (Figure 1C). EPA treatment did not alter total levels of AMPK and ACC, suggesting that EPA-induced phosphorylation of AMPK and ACC was not due to altered expression of these proteins. Since EPA activated AMPK in both BAEC and HUVEC at similar potency (data not shown), we performed most of the experiments in BAEC.

We next examined the dose-dependent effects of EPA on AMPK-Thr172 and ACC-Ser79 phosphorylation. EPA did not affect phosphorylation of AMPK or ACC at a concentration of 5 µM (Figure 1B). In contrast, EPA at 25 µM significantly enhanced AMPK phosphorylation (Figure 1B). Increasing concentrations of EPA (50 and 100 µM) further enhanced AMPK phosphorylation. The changes in ACC phosphorylation mirrored those of AMPK. Levels of total AMPK and ACC remained unchanged at all EPA concentrations tested. Based on these results, 25 µM appears to be the lowest effective concentration of EPA. Thus, BAEC were stimulated with 25 µM EPA for 24 h in subsequent experiments.

### EPA-induced eNOS phosphorylation is AMPK-dependent

**Figure 2:**
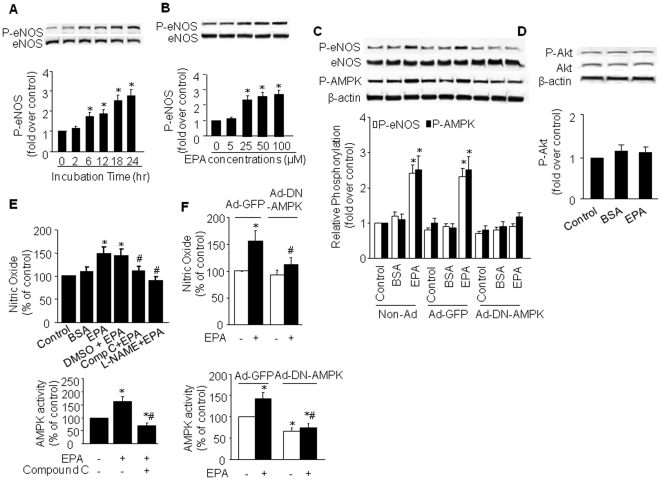
AMPK mediates EPA-induced eNOS phosphorylation and NO production in BAEC. BAEC were treated with (**A**) EPA 25 µmol/L for the indicated times or (**B**) varying concentrations of EPA for 24 h. Lysates were analyzed by western blot for the indicated proteins. The blot is a representative of four blots obtained from four separate experiments. **C**) Western blot of phosphorylated AMPK and eNOS in EPA-stimulated BAEC infected with adenoviruses encoding GFP or Ad-DN-AMPK. **D**) Phosphorylation of Akt in EPA-stimulated BAEC. The data in C and D represent results of 3 separate experiments. For A–D, corresponding densitometric analyses are shown. *P<0.05 *vs.* control. **E**) NO release in EPA-stimulated BAEC. BAEC were treated with compound C (AMPK inhibitor) (20 µmol/L), DMSO (vehicle), or _L_-NAME (NOS inhibitor) (0.1 mM) for 30 min prior to stimulation with EPA. *n* = 4 for each treatment group. *P<0.05 *vs*. control; ^#^ *P*<0.05 *vs*. EPA. **F**) NO release by EPA-stimulated BAEC infected with Ad-DN-AMPK (50 multiplicities of infection) or Ad-GFP (control). *P<0.05 *vs*. non EPA-treated, Ad-GFP group; ^#^ *P*<0.05 *vs*. EPA-treated, Ad-GFP group. For A and B, the corresponding AMPK activity is shown in the lower panel.

We had previously demonstrated that AMPK phosphorylates and activates endothelial nitric oxide synthase (eNOS) in cultured endothelial cells [23]. Similarly, Zhang *et al.* [28] demonstrated that infection of endothelial cells with a recombinant adenovirus expressing the constitutively active AMPK results in eNOS activation and increased NO production. Treatment of BAEC with EPA increased eNOS-Ser1179 phosphorylation, with the time-course of phosphorylation being very similar to that for AMPK phosphorylation (Figure 2A). The dose-dependent effects of EPA on eNOS phosphorylation were also similar to those for AMPK phosphorylation (Figure 2B). Given that EPA activates both AMPK and eNOS in BAEC, we then investigated whether the EPA-stimulated eNOS phosphorylation involves AMPK by infecting BAEC with adenovirus encoding dominant negative AMPK (Ad-DN-AMPK). As expected, treatment of control BAEC (Ad-GFP-infected or non-infected BAEC) with 25 µM EPA for 24 h elicited increased phosphorylation of both AMPK and eNOS (Figure 2C). In contrast, overexpression of Ad-DN-AMPK completely abolished EPA-induced eNOS Ser1179 (equal to Ser1177 in human) phosphorylation.

Both Akt and AMPK are capable of phosphorylating eNOS at Ser1179 [29], prompting us to determine whether Akt may also contribute to EPA-enhanced eNOS phosphorylation. As shown in Figure 2D, EPA did not increase basal Akt phosphorylation at Ser473, suggesting that EPA-stimulated eNOS phosphorylation does not require Akt but depends on activation of AMPK.

### EPA-enhanced NO production is AMPK-dependent

Next, we determined if EPA-induced eNOS phosphorylation is associated with increased NO release. EPA significantly increased NO release, which was inhibited by _L_-NAME, a non-selective inhibitor of NOS (Figure 2E). In addition, inhibition of AMPK with compound C or through infection with Ad-DN-AMPK ablated EPA-enhanced NO release (Figure 2E and F). Importantly, both treatments reduced AMPK activity in EPA-treated cells to below control levels (Figure 2E and F).

### UCP-2 is required for EPA-induced AMPK phosphorylation

**Figure 3:**
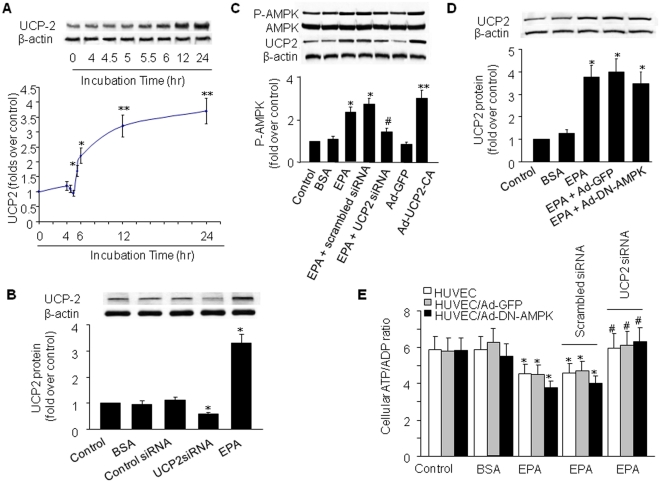
EPA induces AMPK phosphorylation through UCP-2-dependent ATP inhibition. **A**) Time course of EPA upregulation of UCP-2. BAEC were incubated with 25 µM EPA for various amounts of time. After the appropriate incubation time, cells were lysed and UCP-2 protein levels were measured as described. n = 4, *P<0.05; **P<0.01 vs. 0 h time point. **B**) UCP-2 siRNA significantly suppressed basal UCP-2 protein levels. HUVEC were incubated with UCP-2-specific siRNA, scrambled siRNA, EPA and its vehicle BSA for 24 h followed by analysis of UCP-2 protein levels. **C**) Western blot analysis of AMPK and UCP-2 in EPA-stimulated HUVEC. HUVECs were pretreated with UCP-2-specific siRNA (1 µg/30-mm dish), scrambled siRNA, or siRNA transfection reagent. Alternatively, a subset was infected with Ad-CA-UCP-2 or Ad-GFP (control) prior to stimulation. The blot is a representative of four blots obtained from four separate experiments. **D**) Western blot analysis of UCP-2 in EPA-stimulated HUVEC infected with Ad-DN-AMPK (50 multiplicities of infection). Cells left non-infected (non-ad) or infected with Ad-GFP served as controls. The data represent results of three separate experiments. For C and D, corresponding densitometric analyses are shown. **E**) Intracellular ATP/ADP ratio in EPA-stimulated cultures pretreated with scrambled or UCP-2-specific siRNA. Results are expressed as mean ± SD from three independent experiments conducted in triplicate. *P<0.05; **P<0.01 *vs.* control; ^#^ *P*<0.05 *vs.* EPA/scrambled siRNA.

AMPK is activated by an increase in the AMP/ATP ratios and by ATP depletion [30]. Because intracellular ATP production rests mainly on mitochondrial ΔΨ [31], we tested the effect of EPA on the expression of the mitochondrial proton carrier, uncoupling protein 2 (UCP-2). UCP-2 levels were quantified in BAEC treated with 25 µM of EPA for up to 24 h. The increase in UCP-2 protein occurred by 5.5 h and appeared to reach a plateau by 24 h (Figure 3A).

We next determined if genetic inhibition of UCP-2 altered EPA-induced AMPK activation. Since siRNA for bovine UCP-2 was not available, we performed these experiments in HUVEC. As shown in Figure 3B, transfection of UCP-2 siRNA but not scrambled siRNA markedly reduced the basal levels of UCP-2 in HUVEC, implying that HUVEC expressed detectable levels of UCP-2 sensitive to UCP-2-specific siRNA. Moreover, transfection of UCP-2-specific siRNA but not scrambled siRNA significantly abolished EPA-induced UCP-2 expression in HUVEC (Figure 3C). Consistent with these results, siRNA-mediated knockdown of UCP-2 abolished EPA-enhanced AMPK phosphorylation, while scrambled siRNA had no effect (Figure 3C). Further, infection of BAEC with adenovirus encoding constitutively active UCP-2 (Ad-CA-UCP-2) significantly increased AMPK phosphorylation (Figure 3C), indicating that UCP-2 expression was able to activate AMPK in BAEC. On the other hand, and infection of HUVEC with Ad-DN-AMPK did not alter EPA-induced UCP-2 upregulation (Figure 3D). These data indicate that UCP-2 might be required for EPA-induced AMPK activation in endothelial cells.

Because AMPK is highly sensitive to small changes in the intracellular ATP/ADP ratio [14], we explored whether EPA induces alterations in ATP/ADP through upregulation of UCP-2. To test this possibility, HUVEC were transfected with UCP-2 siRNA or control siRNA, then treated with 25 µM EPA for 24 h. Some HUVEC were co-transfected with Ad-DN-AMPK (or with Ad-GFP as a control) to account for slight changes in intracellular ATP levels due to AMPK activation. We found that EPA significantly decreased intracellular ATP/ADP and that UCP-2-specific siRNA blocked this effect in both Ad-DN-AMPK-transfected HUVEC and untransfected HUVEC (Figure 3E). Thus, reductions in cellular ATP levels by EPA may contribute to AMPK activation.

### EPA stimulates UCP-2 expression partly via a PPARγ-mediated pathway

**Figure 4:**
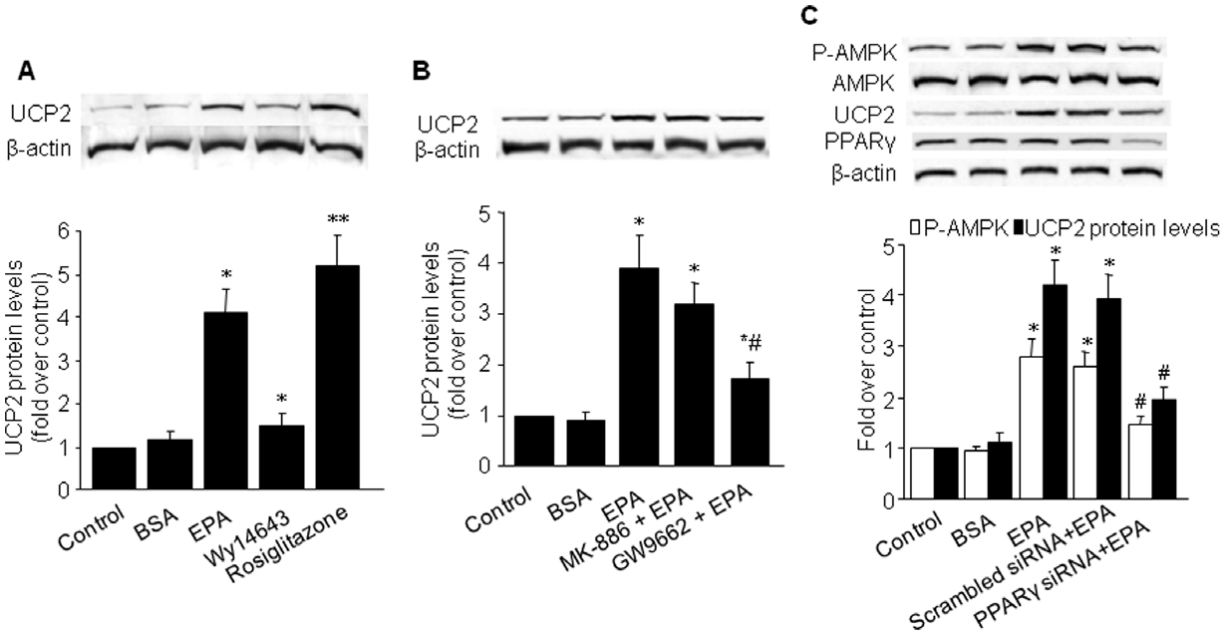
EPA stimulates UCP-2 expression via a PPARγ-mediated pathway in BAEC. **A, B**) Western blot analysis of UCP-2 in EPA-stimulated BAEC pretreated with 10 µmol/L Wy14643 (PPARα agonist), 10 µmol/L rosiglitazone (PPARγ agonist), 10 µmol/L MK886 (PPARα antagonist), or 10 µmol/L GW9662 (PPARγ antagonist). *P<0.05 *vs.* control; **P<0.01 *vs.* control; ^#^ *P*<0.05 *vs.* EPA. **C**) Western blot analysis of AMPK, UCP-2, and PPARγ in EPA-stimulated HUVEC transfected with PPARγ siRNA or scrambled siRNA for 48 h. *P<0.05 *vs.* control; ^#^ *P*<0.05 *vs.* scrambled siRNA + EPA. For A–C, the blot is a representative of four blots obtained from four separate experiments, and the corresponding densitometric analyses are shown.

Thiazolidinediones, which are potent peroxisome proliferator-activated receptor-γ (PPARγ) agonists, have been shown to increase expression of UCP-2 in several tissues [32], leading to the proposal that PPARγ mediates changes in UCP-2 expression [33]. To test whether PPARγ was required for EPA-induced UCP-2 expression, we compared UCP-2 expression among BAEC treated with EPA, Wy14643, or rosiglitazone for 24 h. Rosiglitazone, a PPARγ agonist, elicited a large increase in UCP-2 protein levels (∼5.2-fold, *P*<0.05). This increase was of a slightly greater magnitude to that seen with EPA (Figure 4A). The other PPARα agonist, Wy14643, resulted in a moderate increase in UCP-2 levels (∼1.5-fold, *P*<0.05). Treatment of BAEC with GW9662, an antagonist of PPARγ, partially abrogated the stimulatory effect of EPA on UCP-2 protein expression (Figure 4B), while the PPARα antagonist MK-886 had no effect on UCP-2 expression. These data suggest that EPA-induced upregulation of UCP-2 may be mediated, in part, by PPARγ.

To further verify that EPA increases endothelial UCP-2 expression and AMPK activation through PPARγ-dependent signaling, HUVEC were transfected with PPARγ-specific siRNA and assayed for PPARγ expression, UCP-2 expression, and AMPK phosphorylation in the presence of EPA. Western blotting verified that PPARγ expression was selectively and significantly reduced by the cognate PPARγ siRNA duplex compared with scrambled siRNA (Figure 4C). Importantly, PPARγ siRNA-transfected cells displayed a significant reduction in EPA-induced UCP-2 expression and AMPK phosphorylation compared with mock-transfected cells (Figure 4C).

### EPA induces UCP-2 expression and AMPK α1-mediated eNOS phosphorylation *in vivo*

**Figure 5:**
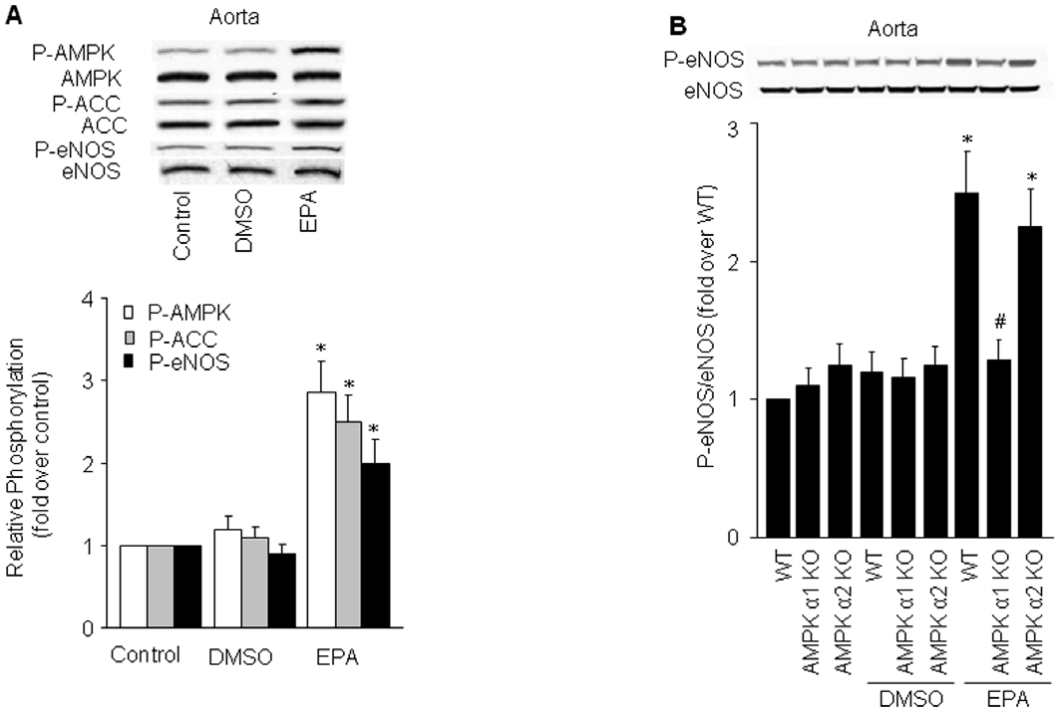
EPA upregulates UCP-2 and activates AMPK in mice. **A**) Western blot analysis of phosphorylated AMPK, ACC, and eNOS in the thoracic aorta of mice receiving EPA or DMSO (vehicle) in their drinking water for 16 weeks (*n* = 3 for each group). Corresponding densitometric analyses are shown. *P<0.05 *vs.* control. **B**) Western blot analysis of eNOS phosphorylation in wild type (WT), AMPKα1 knock out (KO), and AMPKα2 KO mice receiving EPA for 16 weeks (*n* = 3 for each group). The relative p-eNOS/eNOS ratios are shown. *P<0.05 *vs.* non-treated WT; ^#^ *P*<0.05 *vs.* EPA-treated WT. *P<0.05 *vs.* control; **P<0.01 *vs.* control.

To determine whether EPA activates AMPK in vasculature, C57BL/6J mice were given EPA (500 mg/kg/d in drinking water) for 4 months, and aortic levels of AMPK, ACC, and eNOS phosphorylation and UCP-2 protein expression were determined. As shown in Figure 5A, levels of Thr172-phosphorylated AMPK were significantly increased in EPA-treated animals. The phosphorylation of ACC and eNOS was also elevated to a similar degree as that for AMPK.

We also investigated if AMPK mediates the effects of EPA in an isoform-specific manner. As demonstrated in Figure 5B, administration of EPA for 4 months significantly increased the levels of Ser1177-phosphorylated eNOS in aortas from WT and AMPKα2 KO mice. However, EPA activation of eNOS phosphorylation was not observed in AMPKα1 KO animals, indicating that EPA-induced eNOS phosphorylation is mainly mediated by AMPK α1.

### Role of AMPK in EPA-enhanced endothelium-dependent vasorelaxation in Apo-E^−/−^ mice aortas

**Figure 6:**
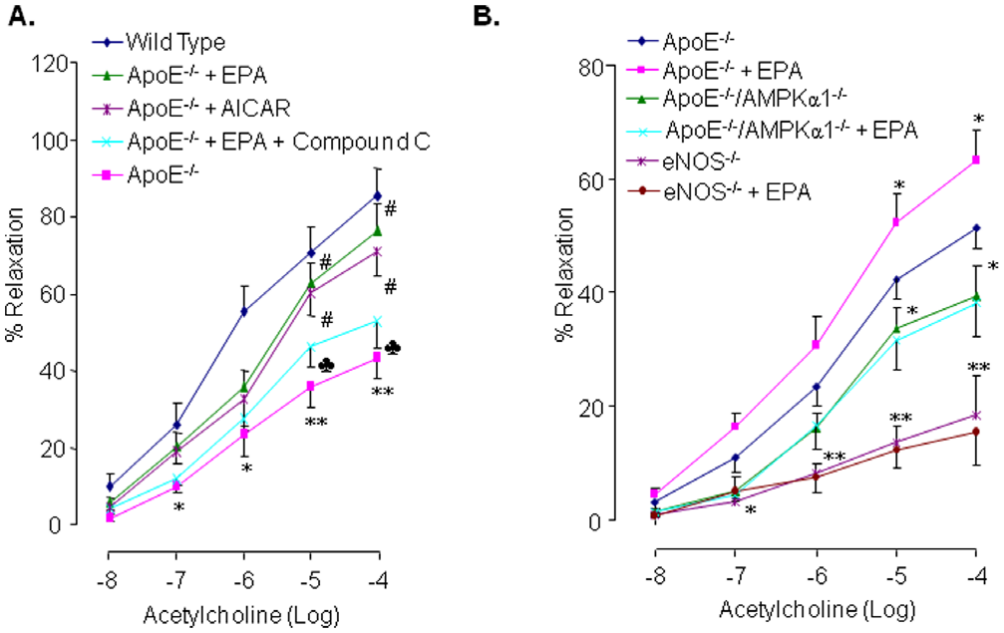
Both AMPK *α1* and NO are required for EPA-enhanced endothelium-dependent vasodilatation *in ex vivo* or *in vivo*. **A**) Endothelium-dependent relaxation of the aortic rings in response to acetylcholine (Ach) from wild type or Apo-E^−/−^ mice. Aortic rings were pretreated ± compound C, then incubated with EPA or AICAR (*n* = 4 for each group). Each data point represents relaxation expressed as a percentage of the value obtained for phenylephrine-preconstricted aorta. *P<0.05 *vs.* wild type; **P<0.01 *vs.* wild type; ^#^ *P*<0.05 *vs.* Apo-E^−/−^; *P*<0.05 *vs.* Apo-E^−/−^ + EPA. **B**) AMPKα1 and eNOS are required for EPA-induced amelioration of endothelium function. Aortic rings extracted from Apo-E KO, Apo-E/AMPKα1 dual KO or eNOS KO mice were incubated with or without EPA (25 µM) for 24 h in EBM. The endothelium-dependent relaxation were assayed by the addition of acetylcholine at concentrations indicated (*n* = 4 for each group). *P<0.05; **P<0.01 vs. Apo-E^−/−^ mice.

Next, to investigate the role of AMPK in endothelial function, we tested the effect of EPA, AICAR, and compound C on endothelium-dependent vasoreactivity under *ex-vivo* conditions. Acetylcholine (Ach) induced concentration-dependent arterial vasodilatation in all groups (Figure 6A). Ach-induced vasodilatation was markedly attenuated in Apo-E KO mice, with the maximum relaxation response of arteries being 44.2±5.4% and that from wild type mice being 85.6±10.5% (*n* = 4 per group, *P*<0.01). It should be noted that, following treatment with AICAR or EPA, Ach-induced vasodilatation in aortas from Apo-E KO mice was significantly improved. In contrast, inhibition of AMPK with compound C abolished EPA-stimulated increases in Ach-induced relaxation in this group, where the maximal relaxation was 76.4±5.5% for EPA alone and 52.9±7.3% in the presence of compound C (Figure 6A). These data suggest that AMPK might play an important role in enhanced endothelial function elicited by EPA.

To further substantiate the role of AMPKα1 in EPA-induced improvement in endothelium function, we assayed the effects of EPA in endothelium -dependent and –independent vasorelaxation in aortas isolated from Apo-E or Apo E/AMPKα1 dual KO mice. As illustrated in Figure 6B, the endothelium-dependent relaxation in response to Ach was significantly reduced in the aortas of Apo-E/AMPKα1 dual KO mice when compared with those of Apo-E KO mice. Further, EPA significantly increased Ach-induced endothelium-dependent relaxation in Apo-E KO but not in the aortas from Apo-E/AMPKα1 dual KO mice (Figure 6B), suggesting that AMPKα1 was required for EPA-enhanced endothelium-dependent vasorelaxation ex vivo.

To further determine the contribution of eNOS in EPA-enhanced relaxation, we next determined the effect of EPA in the aortas from eNOS KO mice. As expected, endothelium-dependent relaxation in response to Ach was minimal (Figure 6B). In addition, EPA at 25 µM did not alter Ach-induced relaxation in the aortas from eNOS KO mice. As endothelium-independent vasorelaxation caused by sodium nitroprusside were similar among Apo-E KO, eNOS KO, and Apo-E/AMPK α1 KO (data not shown). Overall, our results implied that eNOS was required for EPA-enhanced endothelium-dependent relaxation at the doses tested.

## Discussion

AMPK is activated by a rise in AMP and a decrease in ATP, both of which occur by inhibiting ATP production or accelerating ATP consumption [14]. The uncoupling proteins (UCP1, UCP-2, and UCP3) are mitochondrial transporters that are capable of dissipating the proton gradient and increasing thermogenesis, while reducing the efficiency of ATP synthesis [34]. Since AMPK is potently stimulated by depletion of intracellular ATP (or increased AMP:ATP) resulting from the upregulation of UCP-2 in target tissues [35], a possible functional link between these two intracellular systems has emerged. In the present study, we have for the first time provided evidence that EPA via UCP-2 expression increases NO release and endothelial function via AMPK activation in vivo. The beneficial effects of EPA appear independent of serum lipids and EPA via AMPK activation exerts a direct vasoprotective effect. Furthermore, we have characterized that UCP-2 mediates AMPK responses to EPA in endothelial cells. Indeed, our data reveal that EPA significantly increases UCP-2 protein expression and AMPK phosphorylation both *in vitro* and *in vivo*. The upregulation of UCP-2 by EPA is consistent with the findings reported by Armstrong *et al.* [36] Moreover, all classes of unsaturated FFA and/or their metabolites have been shown to upregulate UCP-2 mRNA in cultured cells [37], [38]. Indeed, siRNA-mediated knockdown of UCP-2 protein blocked EPA stimulation of AMPK phosphorylation, and overexpression of UCP-2 directly increased AMPK phosphorylation. These findings support a role for UCP-2 in EPA-induced activation of AMPK.

The mechanism by which EPA increases UCP-2 expression remains unclear. We observed a significant increase in UCP-2 expression after exposure to EPA or PPARγ agonist (rosiglitazone), while only modest increases in UCP-2 expression were observed with the PPARα agonist (Wy14643). The PPARγ antagonist (GW9662) but not the PPARα antagonist (MK-886) significantly blocked EPA-induced upregulation of UCP-2. These results could be further substantiated using specific PPARγ siRNA. Together, these data suggest that EPA induction of UCP-2 expression was mediated partly by PPARγ.

Another important observation of the present study is that chronic EPA supplementation significantly increased eNOS Ser1177 phosphorylation, NO release, and Ach-induced endothelium-dependent relaxation. Further, we have provided evidence that AMPK was required for increased NO bioactivity. Finally, we have shown that AMPK might have direct vaso-protective effects as EPA supplementation improves endothelial function without altering lipids in serum. Our results strongly imply that a direct activation of AMPK in endothelial cells might have broad physiological effects, leading to improvement of endothelial function. In addition to activating eNOS, activated AMPK increases fatty acid oxidation by phosphorylating and inhibiting ACC, which serves to decrease the concentrations of malonyl-CoA [39]. Decreased malonyl-CoA could, in turn, inhibit the accumulation of lipids associated with endothelial dysfunction, which is the precursor of atherosclerosis [40]. Activation of AMPK also decreases fatty acid incorporation into glycerolipids, either secondary to its effect on fatty acid oxidation or through its ability to phosphorylate and inhibit sn-glycerophosphate acyltransferase, the first committed enzyme in diacylglycerol and triglyceride synthesis [41]. An additional benefit of endothelial AMPK activity is that it may inhibit glycerol-3-phosphate acyltransferase, which is required for the *de novo* synthesis of diacylglycerol [41]. In this way, AMPK may lessen endothelial diacylglycerol production (and thus PKC activation) by diminishing availability of the FFA substrate for its synthesis and by directly inhibiting the enzyme which catalyzes its synthesis. The importance of AMPK in the development of endothelial dysfunction and atherosclerosis is best demonstrated by recent studies showing that metformin, one of the most used anti-diabetic drugs which was recently to exert its therapeutic effect in diabetes by activating AMPK [42], [43], has been shown to improve vascular functions and to dramatically reduce cardiovascular endpoints and mortality for type II diabetic patients in large scale clinical trials [44], [45], [46].

In summary, we have uncovered a novel pathway by which the ω-3 fatty acid EPA activates AMPK in endothelial cells. This pathway, which relies on UCP-2 as a mediator of AMPK activation, stimulates NO production through eNOS phosphorylation. Thus, AMPK activation may help account for the beneficial effects of fish oil on endothelial function and atherosclerosis.

## Materials and Methods

### Materials

Bovine aortic endothelial cells (BAEC) and cell culture media were obtained from Clonetics Inc. (Walkersville, MD). Human umbilical vein endothelial cells (HUVEC) and cell culture media were purchased from Cascade Biologics (Portland, OR). FFA-free bovine serum albumin (BSA), palmitic acid, oleic acid, GW9662, and MK-886 were obtained from Sigma (St. Louis, MO). Wy14643 and rosiglitazone were obtained from Cayman Chemical Co. (Ann Arbor, Michigan, USA), and 5-aminoimidazole-4-carboxamide-1-β-D-ribofuranoside (AICAR) was purchased from Toronto Research Chemicals, Inc (Toronto, Canada). Antibodies against phospho-acetyl-CoA carboxylase (ACC) (Ser79), phospho-AMPK (Thr172), AMPK, and phospho-eNOS (Ser1177) were purchased from Cell Signaling Inc. (Beverly, MA). The antibodies against ACC were obtained from Alpha Diagnostic International, Inc. (San Antonio, TX). All other chemicals and organic solvents were of the highest grade and were obtained from Sigma.

### Animals

To generate Apo-E/AMPK dual knockout (KO) mice, Apo-E KO mice obtained from Jackson Labs were crossbred for at least 5 generation with the mice deficient of AMPK α1. Deficiency of both Apo-E and AMPK α1 was confirmed by both RT-PCR and western blots by using the specific antibody against AMPK α1. As both AMPK KO mice and Apo-E KO had extensively been backcrossed to the C57BL6 background, C57BL6 mice aged 8-months were used as wild type control (WT) in the study. All animal protocols were approved by the Institutional Animal Care and Use Committee at University of Oklahoma Health Sciences Center.

### Cell culture, addition of EPA and adenoviral infection

BAEC were grown in EBM supplemented with 2% fetal bovine serum (FBS). HUVEC were maintained in Medium 200 supplemented with a low serum growth supplement (LSGS) before use. All culture media were supplemented with both penicillin (100 Units/ml) and streptomycin (100 µg/ml). Cells between passages 5 and 10 were used for all experiments. All cells were incubated in a humidified atmosphere of 5% CO_2_/95% air at 37°C. The fatty acids were added to the cell cultures coupled to fatty acid-free BSA in the ratio of 2 mol of fatty acid to 1 mol of albumin [47]. When the ECs were 90% confluent, the maintenance medium was removed and cells were treated with EPA (0 to 100 µmol/L), in medium with 2% FCS for 0 to 24 h. In other studies, BAEC or HUVEC were infected with adenoviruses encoding green fluorescence protein (GFP) as a control (Ad-GFP), constitutively active human UCP2 (Ad-CA-UCP2), or a dominant negative mutant form of AMPK alpha (Ad-DN-AMPK). The AMPK-DN adenoviral vector was constructed from AMPK bearing a mutation altering lysine 45 to arginine (K45R) as described previously [42], [48]. Infection was performed in 80% confluent cultures with media containing 0.1% FCS and recombinant adenovirus at a multiplicity of infection of 50. Cells were further incubated with EBM for additional 24 h (HUVEC) or 48 h (BAEC) before experimentation. Using these conditions, infection efficiency was typically at least 80%, as determined by GFP expression.

### SiRNA silencing of PPARγ or UCP2 in HUVEC

HUVEC (passages 3–5) were grown in antibiotic free-EGM-2 medium containing 2% FBS until 70% confluence and transfected with human-specific PPARγ siRNA, UCP2 siRNA, or corresponding scrambled siRNA for 48 h using Lipofectamine™ 2000 (Invitrogen) according to the manufacturer's instructions. The final concentration of siRNA was 200 nM.

### Measurement of nitric oxide production

For NO detection, BAEC grown in 24-well plates were incubated for 30 min in the presence of 15 µM 4,5-diaminofluorescein diacetate (DAF-2 DA) in PBS or in PBS alone (control) in the dark at 37°C. Cells were then washed with PBS to remove excessive DAF-2 DA, and the change in fluorescence was recorded for 15 min at room temperature using a microplate reader (FL 600, Bio-Tek) with the excitation wavelength set at 485 nm and the emission wavelength set at 530 nm. Changes in fluorescence were also visualized with a fluorescence microscope (Olympus IX71), and images were captured for analysis [49].

### Determination of adenine nucleotides

HUVEC were cultured in 6-well plates with control siRNA or UCP2 siRNA for 48 h, treated with EPA or vehicle for 24 h, washed with PBS, and scraped in 0.3 ml of PBS. ATP and ADP were then measured in quadruplicate by a luminometric method as described elsewhere [50].

### Western blot analysis

BAEC or HUVEC and thawed mouse aortas were lysed in cold RIPA buffer. Protein concentrations were determined with a bicinchoninic acid (BCA) protein assay system (Pierce, Rockford, IL). Proteins were subjected to Western blots using ECL-Plus, as described previously [42]. Relative PPARγ protein expression was measured in nuclear extracts from HUVEC as previously detailed [51]. The intensity (area×density) of the individual bands on Western blots was measured by densitometry (model GS-700, Imaging Densitometer; Bio-Rad). The background was subtracted from the calculated area.

### AMPK activity assay

AMPK activity was assayed using the SAMS peptide, as previously described [48]. The activity was determined in the presence and absence of AMP (200 µM). AMPK activity was calculated by determining the difference in activity between both conditions.

### Measurement of endothelium-dependent and endothelium-independent vasorelaxation

Aortic rings (3–4 mm in length) extracted from C57BL6 aged 8-weeks, 20-weeks, 4-months or 8-months (wild type, WT), Apo-E KO aged 8-months, Apo-E/AMPK dual KO mice (8-month old) or eNOS KO (8-week old) were further incubated with EPA (25 µM) or AICAR (2 mM) in endothelial cell basal media (EBM) for 24 h. After that, aortic rings were pre-constricted with phenylephrine in organ chambers (PowerLab, AD Instruments, Colorado Springs, CO). Endothelium-dependent and independent vasodilation responses were determined in the presence of acetylcholine (0.01 to 100 µM) and SNP (0.0001 to 1 µM), respectively. When indicated, compound C (20 µM) were added 30 min prior to addition of EPA.

### Statistics

Statistical comparisons of vasodilation were performed using a two-way ANOVA. Intergroup differences were analyzed using Bonferroni's post test. Analysis of time-course studies was performed with repeated measures ANOVA. All other results were analyzed with one-way ANOVA. Values are expressed as mean ± SD. P values less than 0.05 were considered as significant.
